# P-2021. Implementation of Methicillin-Resistant Staphylococcus aureus PCR Nasal Screening and its' Impact on Antimicrobial Stewardship

**DOI:** 10.1093/ofid/ofaf695.2185

**Published:** 2026-01-11

**Authors:** Edward Lovering, Nadine Safi, Karen McCann

**Affiliations:** MedStar Georgetown University Hospitals Baltimore, Baltimore, MD; MedStar Georgetown University Hospitals Baltimore, Baltimore, MD; MedStar Union Memorial Hospital, Baltimore, Maryland

## Abstract

**Background:**

Methicillin-resistant Staphylococcus aureus (MRSA) nasal screening has a high negative predictive value and supports early de-escalation of intravenous (IV) MRSA-targeted antibiotics. Amid an ongoing IV fluid shortage, optimizing antibiotic stewardship is increasingly important. This quality improvement initiative evaluated the impact of MRSA polymerase chain reaction (PCR) screening on antibiotic de-escalation and IV antibiotic use compared to conventional culture-based screening.

**Methods:**

Between October and December 2024, MRSA PCR nasal screening was implemented for patients admitted to the hospital and receiving IV MRSA-targeted antibiotics. Screening was performed at provider discretion, mirroring real-world clinical practice. Outcomes were compared to a pre-implementation cohort that underwent MRSA nasal culture screening. The primary outcome was the number of IV MRSA-targeted antibiotic doses saved with PCR versus culture. Secondary outcomes included time from specimen collection to result availability.

**Results:**

Among 100 patients who underwent MRSA nasal PCR screening, the most common indications were pneumonia (49%), sepsis of unknown origin (17%), and cellulitis (13%). MRSA PCR was positive in 19 patients and negative in 81. The median time to result was shorter with PCR (12.3 hours [IQR: 7.3]) compared to culture (33 hours [IQR: 4.8]). Among 81 patients with a negative MRSA PCR, IV MRSA-targeted therapy was already discontinued in 11 patients prior to result availability. Of the remaining 70, therapy was de-escalated in 45 patients (64.3%) based on PCR results. The PCR group received 58 fewer doses of IV MRSA targeted antibiotics compared to the pre-implementation culture group.

**Conclusion:**

Implementation of MRSA PCR screening was associated with a substantial reduction in time from collection to result and a meaningful increase in early antibiotic de-escalation, enhancing antimicrobial stewardship efforts. These findings are particularly relevant amid a national IV fluid shortage. This initiative supports broader adoption across hospital units to improve patient outcomes and optimize resource utilization. Future efforts should focus on evaluating the cost-effectiveness of PCR screening and developing standardized criteria for its use.

**Disclosures:**

All Authors: No reported disclosures

